# Acute Stress Dysregulates the LPP ERP Response to Emotional Pictures and Impairs Sustained Attention: Time-Sensitive Effects

**DOI:** 10.3390/brainsci5020201

**Published:** 2015-05-20

**Authors:** Rima A. Alomari, Mercedes Fernandez, Jonathan B. Banks, Juliana Acosta, Jaime L. Tartar

**Affiliations:** Department of Psychology and Neuroscience, Nova Southeastern University, Ft. Lauderdale, FL 33314, USA; E-Mails: ra714@nova.edu (R.A.A.); mf934@nova.edu (M.F.); jb2676@nova.edu (J.B.B.); juliacos@nova.edu (J.A.)

**Keywords:** acute stress, emotion, ERP, late positive potential, sustained attention

## Abstract

Stress can increase emotional vigilance at the cost of a decrease in attention towards non-emotional stimuli. However, the time-dependent effects of acute stress on emotion processing are uncertain. We tested the effects of acute stress on subsequent emotion processing up to 40 min following an acute stressor. Our measure of emotion processing was the late positive potential (LPP) component of the visual event-related potential (ERP), and our measure of non-emotional attention was the sustained attention to response task (SART). We also measured cortisol levels before and after the socially evaluated cold pressor test (SECPT) induction. We found that the effects of stress on the LPP ERP emotion measure were time sensitive. Specifically, the LPP ERP was only altered in the late time-point (30–40 min post-stress) when cortisol was at its highest level. Here, the LPP no longer discriminated between the emotional and non-emotional picture categories, most likely because neutral pictures were perceived as emotional. Moreover, compared to the non-stress condition, the stress-condition showed impaired performance on the SART. Our results support the idea that a limit in attention resources after an emotional stressor is associated with the brain incorrectly processing non-emotional stimuli as emotional and interferes with sustained attention.

## 1. Introduction

Acute stress activates a variety of adaptive responses in the brain and body of an organism. These processes principally occur through the activity of two interrelated biological systems—the autonomic nervous system (ANS) and the hypothalamic-pituitary-adrenal (HPA) axis. Stress-induced responses of the ANS and the HPA axis result in a release of catecholamines and glucocorticoids into the blood stream, respectively.

Initially following a stressor, a surge in ANS catecholamine release induces a state of hyper-vigilance where an individual prioritizes the detection and processing of emotional or stress-relevant stimuli [1,2,3,4,5,6]. However, this post-stress reallocation of attention resources towards relevant stimuli comes at the cost of a reduction in general cognitive processing. For example, following stress, there is an immediate and sustained decrease in prefrontal cortex (PFC) processing [7] leading to impairments in attention towards non-relevant stimuli [6,8,9] and decreases in working memory performance [10]. This increased emotional hypervigilance after stress is likely due to enhanced amygdala activation [6,9,11]. A series of studies suggest that higher cortisol levels can potentiate the emotional hypervigilance after stress. Indeed, cortisol has been shown to increase amygdala activity through its interaction with membrane-bound glucocorticoid receptors [12,13]; high cortisol levels are associated with greater amygdala activation to emotional stimuli [14,15]; and cortisol amplifies catecholamine-induced emotional hypervigilance [14,16,17]. Underscoring these findings is the observation that administration of a norepinephrine reuptake inhibitor and hydrocortisone work synergistically in humans to produce a negative response bias in the amygdala [18]. Even independently of norepinephrine, hydrocortisone administration alone produces increased overall emotionality with experiences ranging from euphoria to depression [19]. It is therefore probable that cortisol-mediated late stress effects alter functional connectivity between the amygdala and the PFC to decrease response specificity in the amygdala, which can alter neural processing of threating or relevant stimuli, leading to indiscriminate hypervigilance for a threat [6,20,21,22]. Ultimately, this indiscriminate hypervigilance would result in both threatening and non-threatening stimuli to be similarly processed.

Given that cortisol is released as part of the late stress response, and has a direct impact on emotion processing, it is critical to understand the time-dependent effects of acute stress on emotion processing in humans. To date, however, these time-dependent effects of stress-induced cortisol release on neurophysiological measures of emotion processing are unclear.

In order to answer this uncertainty, we sought to test the effects of acute stress, relative to a no-stress control condition, on subsequent emotion processing across three time points. Our acute stressor was the socially evaluated cold pressor test (SECPT). The SECPT requires hand immersion into ice-cold water while being socially-evaluated and results in increases in self-report indices of stress as well as elevated saliva cortisol concentrations [23]. Since previous research shows impairments in PFC-dependent attention and working memory performance subsequent to acute stress [6,8,9,10] it was critical to demonstrate stress-induced PFC-dependent impairments here as both a manipulation check, to ensure that any potential *decreases* in emotion processing observed here were not due to global decreases in attention processing, and as a way to break up ERP testing sessions with a non-emotional task. To that end, we employed the PFC-dependent sustained attention to response task (SART) [24]. Embedded within the SART, we also assessed task-unrelated thoughts (TUTs) given the possibility that following stress exposure, stress-related thoughts potentially consume the necessary cognitive resources needed to perform a subsequent non-stressor related task and result in decreased performance [25]. Our measure of emotion processing was the late positive potential (LPP) component of the visual ERP. The LPP ERP is established as a sensitive measure of attention to emotionally-charged visual stimuli [26,27,28,29]. Induction of the LPP is thought to serve as a neurobiological correlate of motivated attention to stimuli of adaptive significance (sex, death, *etc.*). In support of this idea, the LPP is shown to be important for the memory formation of emotional events [30]. Simultaneous fMRI and EEG recording show that the LPP is generated by an extensive network of cortical and subcortical regions with some differential activation by picture valence category (positive *vs.* negative) [31]. Finally, previous work has shown that the LPP amplitude is increased in response to negative stimuli following the SECPT stress [32]. In agreement with the idea that late stress effects are likely to alter emotion processing, the LPP response was increased to negative stimuli after a delay of at least 20 min following acute stress exposure [32].

The specific goals of this study were to: (1) test the effects of the SECPT on processing of pictures with neutral, positive, and negative emotional valence; (2) show potential time-dependent effects of acute stress on emotion processing; and (3) determine the extent to which the SECPT also impacted a non-emotional behavioral measure of sustained attention and TUTs. We first hypothesized that, consistent with previous findings, the SECPT stress would result in a significant increase in cortisol with the highest observed concentrations seen in the last saliva collection (30 min post-stress). Since we were specifically interested in uncovering the potential time-dependent effects of acute stress on emotion processing, we tested the effects of acute stress on the LPP ERP at three different time points following acute stress. We predicted that stress effects on the LPP would be most evident 30 min after stress, concomitant with maximal glucocorticoid concentrations. Finally, we tested the idea that, consistent with previous studies, SECPT-induced stress would result in decreased performance on the SART and on rates of TUTs.

## 2. Method

### 2.1. Participants

Thirty-three undergraduate students (24 women; age: *M* = 19.5 years, *SD* = 2.8 years) were recruited from Nova Southeastern University (NSU) for course credit. Participants were asked to refrain from eating or drinking 1 h prior to participation. All participants were right-handed and had normal or corrected-to-normal vision. Testing procedures were carried out according to a protocol approved by the NSU Institutional Review Board (IRB). Exclusion criteria included a history of cardiovascular disease, Reynaud’s syndrome, seizures, frostbite, an open cut on the non-dominant hand, or psychiatric medication.

### 2.2. Procedure

**Figure 1:**
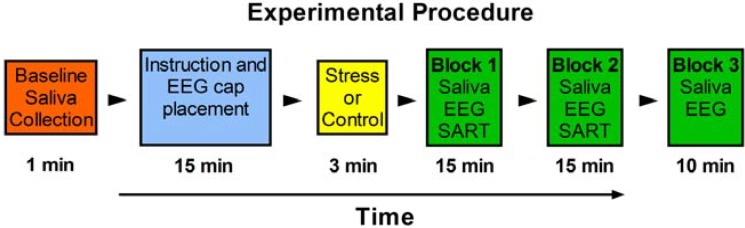
The testing procedure. The testing procedure consisted of 3 testing Blocks after stress (SECPT) or control stress induction.

All testing took place in the afternoon between 2–4 pm in order to control for the circadian variation of cortisol. Participants were first fitted with an electrode cap. Four saliva samples were taken during the study: One as a baseline measure and three additional samples at different time-points after the stressor. A stopwatch was used to ensure consistent timing in the collection of saliva between participants. Following the baseline saliva collection, the participants were exposed to either the SECPT (stress condition) or room temperature water (control (CTL) condition); condition designation (stress *vs.* control) was randomized. As shown in Figure 1, the participants were tested three times (across three blocks of testing) after stress or control exposure. Block 1 occurred 1–15 min after the stress, Block 2 occurred 15–30 min after the stress, and Block 3 occurred 30–40 min after the stress. Blocks 1 and 2 each included a saliva sample, followed by EEG testing, and SART. Together, these within-block activities kept participants actively engaged between saliva samples, which were collected every 15 min. The last saliva sample was taken at the start of Block 3, and was followed by EEG testing. This final block did not include the SART. Within each block, the saliva collection took approximately 1 min, the EEG took approximately 9 min, and the SART took approximately 5 min.

### 2.3. Stimuli and Physiological Recording

#### 2.3.1. SECPT and Control

The temperature of the water for the stress condition (*n* = 19) was maintained at 3 °C and the water temperature of the control condition (*n* = 14) was maintained at 23 °C [33]. In addition, participants in the experimental condition (SECPT) were observed by an unfamiliar and unfriendly experimenter in the room and video-recorded in order to add the social evaluation aspect of the stressor [23]. The participants in the control condition were neither video recorded nor was there an experimenter evaluating them.

#### 2.3.2. Salivary Cortisol Collection

Saliva samples were collected from each participant through passive drool into polyethylene tubes. Immediately after collection, the sample tubes were stored in a −20 °C freezer. For cortisol quantification the saliva samples were thawed, vortexed, and centrifuged at 3000 rpm (0.9× *g*) for 15 min. Samples were then quantified from the thawed saliva sample solution via human enzyme immunoassay (EIA) kits per the manufacturer’s instructions (Salimetrics LLC, Carlsbad, CA, USA). The samples were immediately read in a BioTek ELx800 plate reader (BioTek Instruments, Inc., Vinooski, VT, USA) at 450 nm with a correction at 630 nm. Final concentrations were generated in μg/dL by interpolation from the standard curve using a 4-parameter non-linear regression curve fit.

#### 2.3.3. Emotional Stimuli

Emotional responses were elicited by visual stimuli from the International Affective Picture System (IAPS) (see the Supplementary Information) [34]. The IAPS contains positive and negative pictures that are rated to be high on valence and arousal relative to neutral pictures. There were 105 trials and each trial began with a 400 ms randomized presentation of either a negative (*n* = 35) neutral (*n* = 35) or positive (*n* = 35) IAPS picture followed by 3000 ms of a black screen. The IAPS normative ratings [35] were used to select the emotional category of each picture. Independent picture sets were used for each time-point and the order was counter-balanced across participants. The picture sets were matched on normative valence and arousal ratings for each picture category. The 400 ms picture on-time was chosen since even briefly presented pictures capture attention resources, but also has the advantage of better evoking both early posterior negativity (EPN) and LPP components [36]. Following the picture presentation, a black screen was on for the rest of the trial. All pictures were presented on a 23-inch LCD monitor with a vertical refresh rate of 60 Hz. A central fixation point was present in the center of the screen throughout the experiment. As a manipulation control (to ensure that the participants were attending to the pictures) participants were asked to rate the valence of each picture during the 3000 ms period following picture presentation. The pictures were rated on a 1–9 scale in accord with the normalized scores—1 being most negative and 9 being most positive [35]. Picture presentation and timing were controlled through the use of Presentation software (Neurobehavioral Systems, LLC, Berkeley, USA).

#### 2.3.4. Electrophysiological Recordings

EEG assessment was conducted using Contact Precision Instruments’ Psychlab EEG amplifying and recording equipment (Contact Precision Instruments, Cambridge, MA, USA). EEG activity was recorded with a cap fitted with pure tin cup electrodes at Fz, Cz, Pz, C3, C4, O1, and O2 (Electro-Cap International, Eaton, OH, USA) placed in accordance with the International 10–20 System. Eye movements and eye blinks were monitored via tin electrodes (Electro-Cap International, Eaton, OH, USA) placed above and at the outer canthus of the left eye. Signals were referenced to linked electrodes attached to earlobes. Electrode impedance was maintained at less than 5 kΩ. Procedures for infection control specified by the Society for Psychophysiological Research were followed in attaching and removing electrodes [37]. The EEG amplifier was set at a gain of 30,000 and the sampling rate of the EEG was 500 Hz. High pass filters were set to 1 Hz and low pass filters were set to 40 Hz. A 60 Hz notch filter was active. The data were analyzed offline with Psylab8 software (Contact Precision Instruments, Cambridge, MA, USA). For the ERP analyses, 1000 ms of raw EEG data were epoched to the respective stimulus presentation including a 100 ms pre-stimulus baseline. The LPP was measured as the average voltage between 300–800 ms following picture onset. Trials in which the EOG exceeded ±75 μV were excluded from the final averaged ERP. The ERP trials were also visually examined and individually rejected at each electrode location for any additional observed artifact (e.g., blocking, movement, alpha). The final number of trials analyzed for the control condition was as follows: Neutral pictures mean = 31.29, *SD* = 1.73, positive pictures mean = 30.86, *SD* = 2.07, negative pictures mean = 30.29, *SD* = 1.54. The final number of trials analyzed for the stress condition was as follows: Neutral pictures mean = 30.88, *SD* = 2.32, positive pictures mean = 32.57, *SD* = 2.31, negative pictures mean = 32.42, *SD* = 2.10.

#### 2.3.5. SART Performance

The SART is widely recognized as an effective test for sustained attention [24]. The SART is a go/no-go task, where the go stimulus (non-target) appears and requires a response (e.g., pressing the space bar) and the no-go stimulus (target) appears and requires withholding a response (e.g., not pressing the space bar). Non-target stimuli were digits ranging from 1 to 9, excluding 3, which was the target digit. Each trial included a digit displayed for 250 ms on a grey screen, followed by a fixation cross displayed for 900 ms. Participants could respond during either the digit or fixation cross display. Targets were presented on 11% of trials, for a total of 20 target trials and 160 non-target trials. To measure whether participants experienced TUTs, 12 thought probes were randomly inserted into the SART following target trials (*i.e.*, no-go stimulus). During the probe, participants were asked “What are you thinking about?” Participants responded by selecting one of the following response options: (1) on task only, (2) task performance, (3) negative thoughts, (4) positive thoughts, (5) water task, and (6) other task-unrelated thoughts. Percentage of TUTs was calculated by summing the responses indicated as option 3–6.

#### 2.3.6. Statistical Analyses

An increase in HPA axis activity (cortisol) in response to the SECPT was assessed by mixed-model ANOVA with time as the within-subject factor (four time points) and condition as the between-subject factor (stress *vs.* control). In order to examine the effect of picture category on the visual LPP across electrodes in the stress *vs.* control condition, we carried out a 3 × 3 × 7 mixed-model ANOVA. The within factors were time (Blocks 1–3, see Figure 1), picture category (positive, negative, neutral) and electrode locations (Fz, Cz, Pz, C3, C4, O1 and O2). The effect of stress on the sustained attention task was analyzed with a series of 2 × 2 mixed-model ANOVA on target accuracy, non-target accuracy, and non-target reaction time with time (Blocks 1 and 2, see Figure 1) as the within subjects factor and condition (stress *vs.* control) as the between subjects measure. TUTs during the SART was analyzed by thought probe responses using a 2 (time: Block 1 and Block 2) × 2 (condition: Stress *vs.* control) mixed-model ANOVA with condition as the between subjects factor. To examine the relationship between cortisol and TUTs, correlations were computed between cortisol level and percentage of TUTs immediately following the SECPT and at the last time point (*i.e.*, Block 2).

All calculations were conducted using an SPSS statistical package (version 19, SPSS Inc., IBM Company, Armonk, NY, US). In instances where the sphericity assumption was not met, the reported *p*-values associated with the F statistics were adjusted via Greenhouse-Geisser, and all analyses include a measure of partial η^2^ as effect size. The Bonferroni method was applied to correct for multiple comparisons. All reported p values have an a priori significance level of *p* < 0.05.

## 3. Results

### 3.1. Cortisol

**Figure 2:**
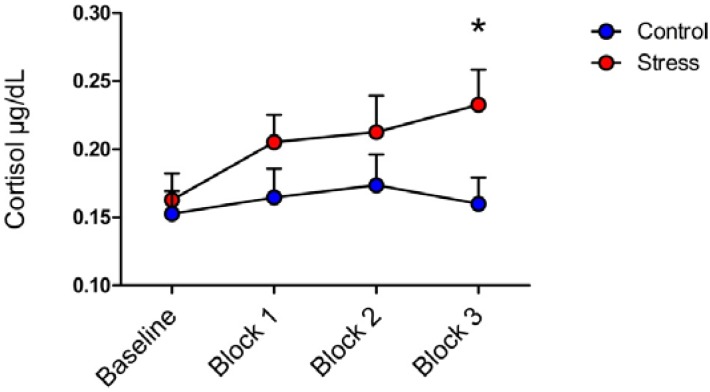
Cortisol levels for the two conditions at four time points. There was a significant time × condition interaction (*F* (3, 93) = 4.22, *p* < 0.05, partial η^2^ = 0.11) for cortisol with the stress condition yielding higher cortisol levels relative to the control condition in Block 3 (*p* < 0.05).

There was a significant time × condition interaction (*F* (3, 93) = 4.22, *p* < 0.05, partial η^2^ = 0.11). To investigate this interaction, independent-samples t-tests were conducted to compare cortisol levels between conditions at each timepoint (baseline and Blocks 1–3). As can be seen in Figure 2, there was a significant difference in the cortisol levels at Block 3 between the stress (*M* = 0.23, *SD* = 0.11) and control (*M* = 0.16, *SD* = 0.07) conditions; *t*(31)= −2.13, *p* = 0.04, *d* = 0.76.

### 3.2. LPP and Picture Analyses

**Figure 3:**
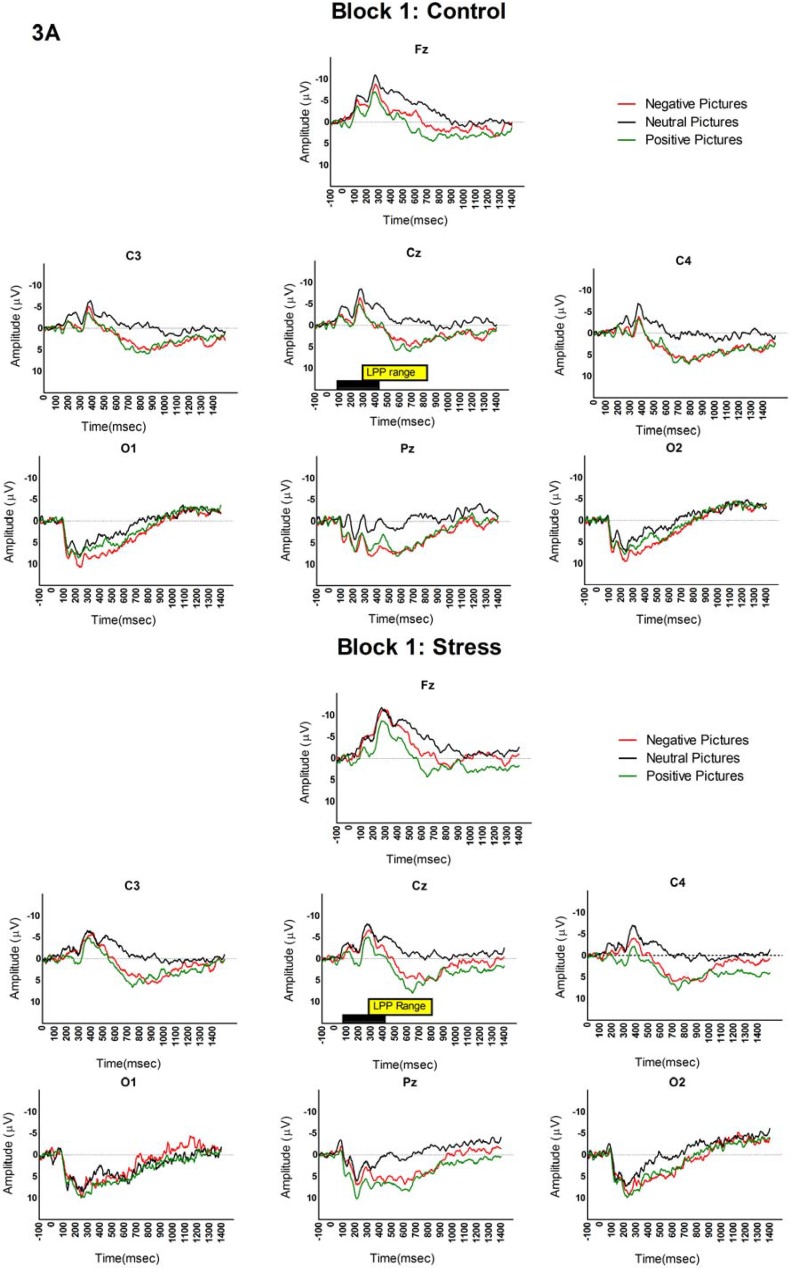

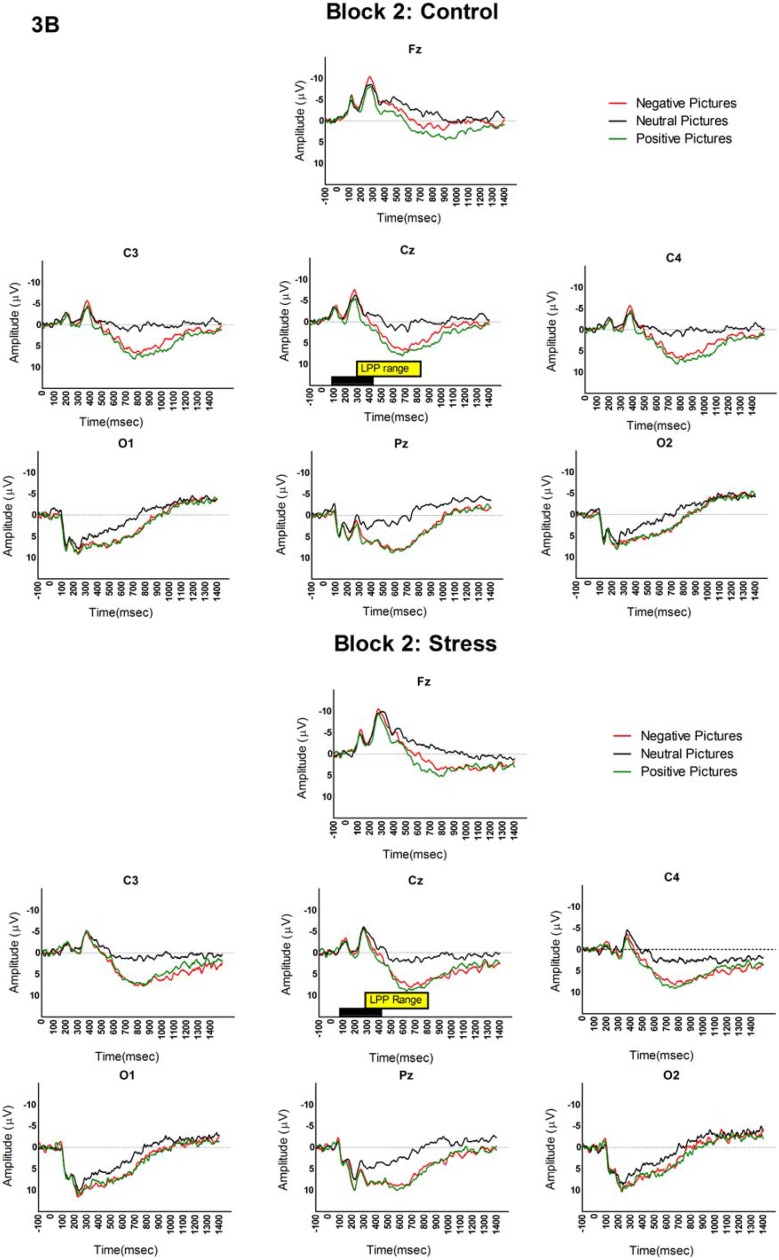

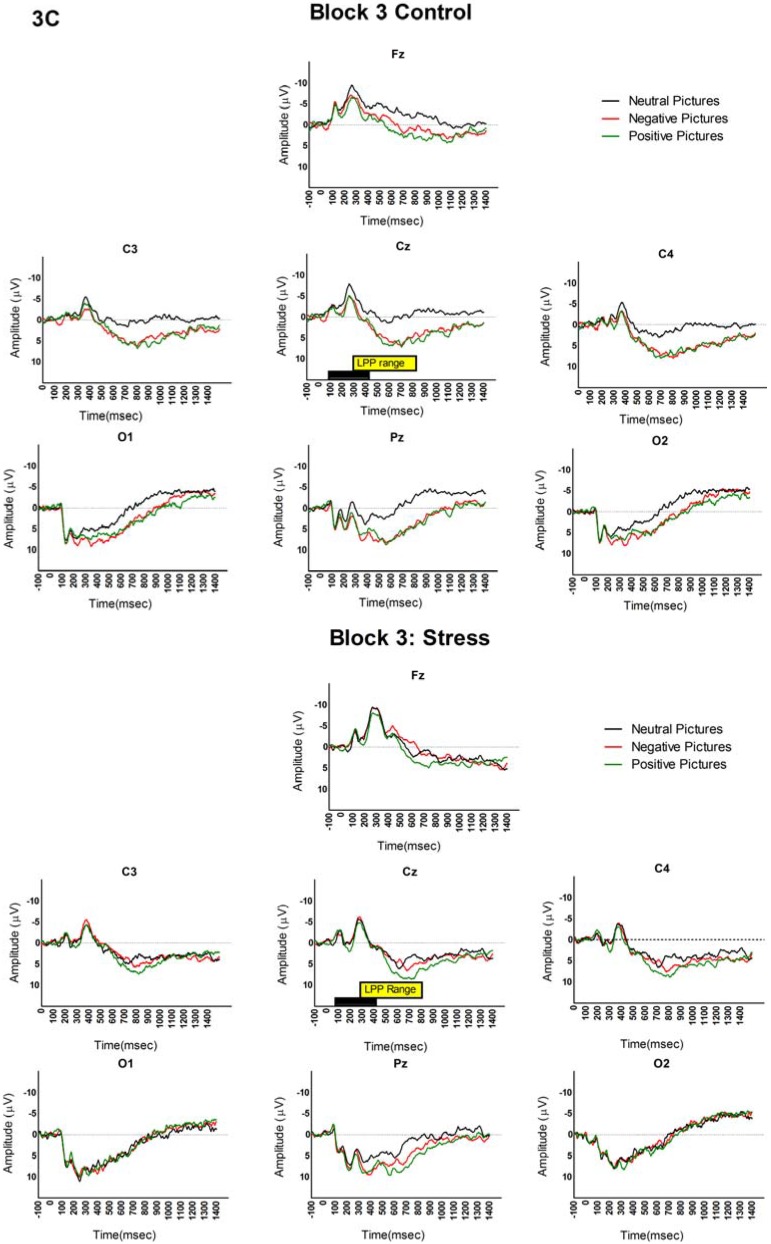
LPP ERP. The LPP ERP elicited by the IAPS in the control and stress conditions during each Block at each electrode location. In Blocks 1 and 2 (**3A**) and (**3B**), there were no differences in the LPP amplitude between the stress and the control conditions. However, in Block 3 (**3C**), the neutral pictures elicited a larger LPP in the stress condition relative to the control condition during Block 3. Black horizontal bar in Cz indicates the 400 ms picture exposure and the yellow horizontal bar indicates the LPP analysis latency range.

Figure 3 presents the grand average visual ERPs separated by time and picture category, for all electrode locations. We analyzed the visual LPP using a 3 (time) × 3 (picture category) × 7 (electrode location) mixed-model ANOVA with condition (stress *vs.* control) as the between subjects factor. As expected and consistent with previous studies, this analysis revealed a significant main effect for picture category (*F* (2, 68) = 29.67, *p* < 0.001, partial η^2^ = 0.49); the LPP responses to negative (*M* = 4.66, *SE* = 0.65) and positive pictures (*M* = 3.84, *SE* = 065) were larger than to neutral pictures (*M* = 0.24, *SE* = 0.71). There was also a significant main effect for electrode location (*F* (6204) = 13.06, *p* < 0.001, partial η^2^ = 0.30), but no main effect of condition, *p* > 0.05. The interaction analyses showed a significant time x electrode interaction (*F* (12,408) = 2.52, *p* < 0.01, partial η^2^ = 0.08) and significant picture × electrode interaction (*F* (12,408) = 11.60, *p* < 0.001, partial η^2^ = 0.27, but no time × condition interaction (*F* (2, 62) = 0.33, *p* = 0.72). Although we did not observe a significant effect of condition or a time × condition interaction, we did observe time × electrode and picture × electrode interactions. To test our a priori hypothesis that the effects of stress on LPP would be altered at the latest post-stress time-point (30–40 min post stress induction), a series of exploratory planned one-way ANOVA were conducted separately at each time-point in order to observe the effects of condition (stress *vs.* control) at each electrode location and for each picture category. This analysis confirmed the visual observation and our expectation that the LPP differentiated between emotional and non-emotional pictures in both the stress and the control condition early on after stress induction (*i.e.*, in Block 1 and 2), such that LPP amplitude for neutral pictures (Block 1, *M* = −0.51, *SE* = 1.09; Block 2, *M* = 0.18, *SE* = 0.83) was lower than negative (Block 1, *M* = 3.45, *SE* = 0.86; *p* < 0.01 ; Block 2, *M* = 4.41, *SE* = 1.05; *p* < 0.05) and positive pictures (Block 1, *M* = 4.02, *SE* = 0.91l *p* < 0.05; Block 2, *M* = 5.21, *SE* = 0.94; *p* < 0.01). No differences were found between the two condition for LPP response to emotional pictures (positive or negative) in Block 3, all *p*’s >0.05. However, as predicted, later stress effects (at Block 3) dysregulated the LPP response, such that there was not a significant effect of picture for the stress condition, *p* > 0.05. Specifically, the LPP for the neutral pictures were significantly larger in the stress condition compared to the control condition at Fz (control *M* = −2.82, *SD* = 6.49; stress *M* = 1.87, *SD* = 6.17; *p* < 0.05), Cz (control *M* = −0.22, *SD* = 5.25; stress *M* = 4.01, *SD* = 4.99; *p* < 0.05), and O1 (control *M* = −0.30, *SD* = 3.68; stress *M* = 2.63, *SD* = 4.24; *p* < 0.05) electrode locations. However, the LPP amplitude for the control condition was similar to response seen in Blocks 1 and 2, with neutral pictures showing a smaller LPP amplitude (*M* = −0.55, *SE* = 1.67) than negative (*M* = 3.45, *SE* = 1.37; *p* < 0.05) or positive pictures (*M* = 3.82, *SE* = 1.14; *p* < 0.05).

### 3.3. Picture Rating

Analyses of the response data to the positive, negative and neutral pictures as a manipulation verification showed that there were no differences in responses between conditions or between time-points (all *p*’s >0.05)—The participants attended to the task and correctly identified the picture categories. The overall average rating of the picture categories were: Positive pictures: *M* = 7.27, *SD* = 1.08; negative pictures: *M* = 2.22, *SD* = 0.96, neutral pictures: *M* = 5.25, *SD* = 0.54.

### 3.4. SART

**Figure 4:**
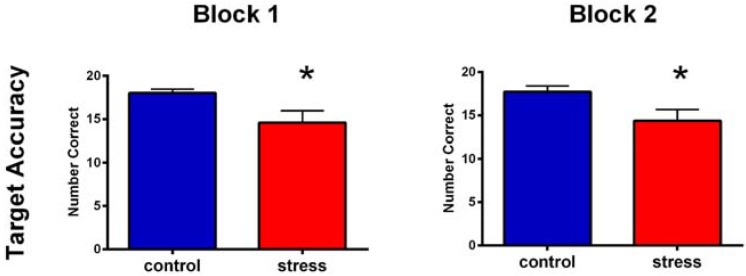
SART performance. There was impaired target accuracy and delayed reaction time on the SART at 15 min and 30 min following stress (black bars represent 1 standard error). Asterisks indicate a statistical difference between stress and control condition at *p* < 0.05.

Figure 4 presents the average of target response accuracy and non-target reaction time, separated by time and condition. We analyzed the target accuracy, non-target accuracy, non-target reaction time, and TUTs using a series of 2 (time) × 2 (condition) mixed-model ANOVA with condition as the between subjects factor. This analysis revealed a significant main effect of condition on target accuracy for SART performance at Block 1 (*F* (1, 31) = 4.19, *p* < 0.05, partial η^2^ = 0.12) and Block 2 (*F* (1, 31) = 4.20, *p* < 0.05, partial η^2^ = 0.12); the stress condition had lower target accuracy (Block 1 *M* = 14.58, *SD* = 6.05 and Block 2 *M* = 14.36, *SD* = 5.67) than the control condition (Block 1 *M* = 18.00, *SD* = 1.75 and Block 2 *M* = 17.71, *SD* = 2.58). No effect of time or time by condition interaction was observed (all *p*s > 0.05). No significant effect of time, condition, or time × condition interaction were observed for non-target accuracy (all *p*s > 0.05). A significant effect of time was found for non-target reaction time (*F* (1, 31) = 11.76, *p* < 0.01, partial η^2^ = 0.27), but no effect of condition or time × condition (all *p*s > 0.05). Reaction time for non-target trials were longer during Block 1 (*M* = 301.12 ms *SD* = 94.41) than during Block 2 (*M* = 271.21 ms *SD* = 78.50).

There was no main effect for condition on TUTs during the SART (*F* (1,31) = 0.94, *p* = 0.34) or a time × condition interaction (*F* (1,31) = 0.61, *p* = 0.44), but a significant time effect (*F* (1,31) = 11.29, *p* < 0.01, partial η^2^ = 0.27) indicated an increase in percentage of TUTs from Block 1 (*M* = 0.23, *SD* = 0.22) to Block 2 (*M* = 0.37, *SD* = 0.28). No significant relationships were observed between any of the SART measures and TUTs in Block 1 or 2, all *p*’s > *0.05*.

To examine the relationship between TUTs and cortisol the correlations between percentage of TUTs (Block 1 and Block 2) and cortisol levels (Block 2 and Block 3) were computed. No significant relationships were observed at any time points or by condition, all *p*’s > 0.05. Based on [12] findings that increased cortisol was related to increases in stressor related thoughts, we examined the percentage of times participants indicated they experienced TUTs related to the stress task (*i.e.*, percentage of thought probe 5 responses). No relationship was found between the percentage of probe 5 thoughts during the SART immediately following the SECPT (*i.e.*, during Block 1) and the cortisol measure immediately following this SART (*i.e.*, at the start of Block 2), *p* > 0.05. A significant relationship was found, however, collapsing across both conditions, between the percentage of probe 5 thoughts during the second SART (Block 2) and the cortisol measure taken immediately following this SART (*i.e.*, at the start of Block 3), *r*(31) = 0.43, *p* < 0.05.

## 4. Discussion

We demonstrate time dependent effects of stress induction on a neurophysiological measure of emotion processing. While the effects of stress on the LPP ERP emotion measure were maintained during Blocks 1 and 2, there was an apparent hypervigilant neural response during Block 3 (30–40 min after stress) in that the LPP ERP no longer discriminated between neutral and emotional pictures. Although we did not observe a time by condition interaction, possibly due to lower power, our planned analysis supported these time dependent effects. Noteworthy, this effect occurred when cortisol levels were at their highest. Moreover, in agreement with previous studies, compared to the control condition, participants in the stress condition performed poorly on target trials of the SART (during both Block 1 and Block 2). Also consistent with previous research, cortisol responses were significantly higher in the stress condition compared to the control condition.

Previous LPP ERP studies on emotion processing have found that, relative to non-emotional pictures, emotional pictures garner greater attention resources [3,36,38,39]. The increase in attention resource allocation for emotional picture processing can be explained in terms of the high intrinsic motivational properties of the picture [40]. In agreement with this idea, we find that at earlier time-points following stress (Blocks 1 and 2), emotional processing necessary for LPP generation was maintained—In spite of reduced PFC-dependent sustained attention task performance at this time. Since the affective stimuli were able to capture attention resources and command priority processing (due to their high motivational relevance) this suggests that natural selective attention mechanisms are preserved for a short time period after stress [1,3,4,34,41]. However, our findings also reveal that stress exposure can impair the ability of the LPP to discriminate between emotional and non-emotional pictures after a delay. This increased amplitude in response to non-emotional pictures is in line with our original hypothesis that stress-induced changes would occur as part of the late stress response. We specifically found that, unlike the earlier blocks, in Block 3 (30–40 min following stress) there was an increased LPP response to non-emotional pictures; probably due to an already heightened emotional state, which was not easily switched off following stress [42,43,44]. In other words, the rapid and unpredictable presentation of emotionally neutral pictures amidst emotional pictures appears to have led to a state of emotional dysregulation where the brain no longer “switched” states and instead maintained an emotional response to all stimuli, which led to the incorrect neural processing of non-emotional stimuli as threatening. That we see this as part of the late stress response and previously observed this after sleep deprivation [45] suggests that the ability to automatically and rapidly categorize emotional and non-emotional pictures is sensitive to perturbations that alter or consume attention resources. These ideas are in agreement with the notion that high levels of cortisol release can amplify the effects of catecholamines on threat detection and amygdala activity [46,47]. While this type of indiscriminate evaluation of stimuli might come at the cost of “false positives” it may also facilitate the detection of real threats to optimize rapid adaptive behavior by relocating neural resources away from higher-order cognitive processing regions in the PFC to limbic structures [48].

This study also expands on previous work which tested the LPP response to emotionally relevant, unpleasant stimuli approximately 30 min following CPT stress induction [32]. We directly build upon this work by showing the early and late stress effects on both pleasant and unpleasant stimuli. Unlike a previous study [32] which showed an increase in the LPP to emotional pictures relative to neutral pictures following stress, our study revealed an increase in the LPP to non-emotional (neutral) pictures. These dissimilarities can potentially be explained by differences in the experimental design of the current study such as the use of positive and negative pictures, a shorter picture on-time (400 ms *vs.* 3000 ms), and exposure to the SART.

Since late stress responses are related to the release of glucocorticoids, cortisol is a viable candidate to observe the temporal dynamics of the stress-induced changes in neural processing. Importantly, however, cortisol is not necessarily the direct mediator of the LPP response. Although cortisol is a marker of the late stress response, we did not find a direct relationship between cortisol and LPP amplitude. Cortisol activity as part of the late stress response recruits, and is co-activated with, a host of many neuromodulators that can each influence stress-induced changes in neural processing. Indeed, a follow-up analysis of cortisol and LPP amplitude showed no correlation between cortisol and LPP amplitude at any electrode location. We also conducted a secondary analysis that separately considered high and low responders (based on cortisol levels) and still did not find a correlation between cortisol level and LPP amplitude. Consequently, we do not believe that cortisol is directly affecting our LPP findings, but rather, both occur as part of the late stress response. This is also in agreement with our previous work where we did not observe any change in cortisol concentrations after sleep deprivation before or after emotional picture exposure, suggesting that the observed changes in emotion processing are independent of potential stress effects of sleep deprivation [45]. In addition, circulating cortisol levels do not necessarily correspond directly to neural cortisol responses due to individual differences in glucocorticoid receptor sensitivity [49,50].

Our SART findings showed that, as expected and consistent with previous studies, sustained attention processes were impaired following stress. These findings add to existing knowledge by showing that subsequent to SECPT stress induction the impairment in sustained attention: (1) is not sensitive to repeated testing; and (2) is present at approximately 15 and approximately 30 min post-stress. The observation that TUTs were not significant predictors of SART performance suggests that the redirection of attention resources toward affective stimuli following stress may occur at a level below metacognitive awareness. In fact the increase in TUTs over time occurred in both conditions but did not result in changes in SART performance.

The current study replicated the relationship between increased cortisol and thoughts related to the stressor [51] but this relationship was observed only during the second testing block. Although the increased LPP to the neutral pictures occurred when cortisol was at its peak post-stress level, we did not experimentally manipulate cortisol levels and thus cannot ascertain whether cortisol served as mechanism though which stress altered the ability of the LPP to discriminate between the emotional and neutral pictures. Nevertheless, since these results suggest that there might be a relationship between cortisol and LPP processing, it will be of interest for future studies to investigate the direct effect of hydrocortisone administration on the LPP response to emotional and non-emotional pictures. Further, since our primary interest in including the SART was as a manipulation check, to break up LPP testing, and as a mechanism to test for general attention effects after stress (in case we observed decreases in emotional attention), we did not include a third SART testing session. For this reason, it is not certain if sustained attention would be altered as part of the late stress effects (in the 3rd Block).

One limitation of the present study is that we did not extend our testing time past the 30–40 min testing block. It is unknown if a time period after 40 min would show heightened responses or a recovery of LPP and sustained attention responses [42,52]. Future studies, should extend the time periods of testing to investigate whether there will be changes in emotion processing; especially as cortisol returns to baseline levels. An additional consideration is that the LPP is typically most robust at central-parietal electrode locations, but we find that the induction of an LPP to the neutral pictures after stress showed a broad scalp distribution and was most robust (reached significance) at frontal (Fz), central (Cz) and occipital locations (O1). Previous work showing experimental LPP effects at frontal electrode locations showed that in response to emotional stimuli, the LPP is most robust at the Fz electrode location [53] when viewing pain and during a reappraisal task in evaluating angry faces [54]. The findings relating the LPP to the reappraisal of angry faces were similar to the current study in that LPP to the emotional visual stimuli was shown to be most robust at the Fz and Cz electrode locations at the 300–600 ms latency range. It is possible that the experimental manipulation in these studies (pain evaluation, reappraisal) and ours (stress exposure) preferentially involve changes to frontal brain region attention processes. A final consideration is that the participants were able to correctly classify the picture categories while the LPP measure did not differentially modulate between picture categories (emotional *vs.* non-emotional). However, these findings agree with previous work by us which showed that the ERP can serve as a more sensitive indicator of neural processing compared to behavioral measures [55,56]. In addition, the timing of the LPP recording was before and for a shorter time window (300–800 ms post picture onset) relative to the behavioral ratings (rated between 400–3000 ms post picture onset). This afforded the participants more processing time to evaluate the valence of the pictures in the behavioral ratings.

## 5. Conclusions

In summary, we examined potential time-dependent effects of acute stress on sustained attention and emotion processing. We found that, consistent with previous reports, acute stress results in a decrease in sustained attention. The ERP LPP response was only affected after 30 min. At this point the LPP appeared to reflect emotional dysregulation as it no longer discriminated between the emotional and non-emotional picture categories due to an apparent perception of the neutral pictures as emotional. We are currently planning a series of follow up experiments aimed at investigating the effects of multiple acute stress conditions on emotional and non-emotional attention, at time points beyond the 30–40 min window, after stress induction, as well as the effect of stimulus presentation length on LPP modulation and behavioral ratings.

## Supplementary Files

Supplementary File 1
