# Supplementary material for: Acute Stress Dysregulates the LPP ERP Response to Emotional Pictures and Impairs Sustained Attention: Time-Sensitive Effects
Source: Brain Sci. 2015 May 20;5(2):201–19. doi: 10.3390/brainsci5020201 (PMC4493465; doi:10.3390/brainsci5020201)
Supplement: Supplementary File 1 [file brainsci-05-00201-s001.pdf]

Supplementary Information

IAPS picture directory reference numbers for the images used in the present study. Neutral picture set 1: 6910, 7002, 7009, 7014, 7019, 7030, 7033, 7036, 7040, 7044, 7052, 7056, 7057, 7061, 7080, 7096, 7130, 7161, 7180, 7184, 7188, 7205, 7207, 7224, 7237, 7247, 7249, 7287, 7354, 7487, 7546, 7500, 7560, 8060, and 8312. Neutral picture set 2: 7000, 7003, 7010, 7017, 7020, 7025, 7031, 7034, 7037, 7041, 7045, 7053, 7058, 7062, 7081, 7100, 7140, 7160, 7170, 7182, 7185, 7187, 7190, 7211, 7235, 7242, 7255, 7290, 7365, 7493, 7506, 7547, 7820, 8065, and 8232. Neutral picture set 3: 7001, 7004, 7012, 7018, 7021, 7026, 7032, 7035, 7038, 7043, 7050, 7055, 7059, 7077, 7090, 7110, 7150, 7175, 7179, 7183, 7186, 7192, 7217, 7233, 7236, 7248, 7285, 7300, 7484, 7497, 7513, 7550, 7830, 8160, and 8192. Negative picture set 1: 1052, 1201, 1271, 2053, 2205, 2276, 2375.1, 2683, 2799, 2800, 3000, 3001, 3005.1, 3010, 3017, 3030, 3060, 3064, 3230, 3400, 6021, 6212, 6260, 6312, 6510, 6530, 6831, 9181, 9290, 9491, 9520, 9561, 9902, 9903, and 9927. Negative picture set 2: 1051, 1202, 1274, 2095, 2455, 2688, 2730, 2811, 2900, 3015, 3016, 3053, 3062, 3063, 3261, 3266, 3301, 3350, 3500, 3530, 6022, 6213, 6250, 6311, 6415, s6550, 6834, 9075, 9184, 9291, 9530, 9600, 9901, 9904, and 9922. Negative picture set 3: 1050, 1220, 1275, 2352.2, 2710, 2750, 2751, 2981, 3059, 3063, 3069, 3300, 3550, 6242, 6243, 6300, 6360, 6560, 6570, 6838, 9075, 9295, 9301, 9405, 9410, 9412, 9413, 9433, 9500, 9560, 9611, 9900, 9908, 9921, and 9940. Positive picture set 1: 1440, 1463, 1500, 1721, 1750, 1999, 2045, 2058, 2080, 2154, 2170, 2260, 2314, 2341, 2360, 2398, 2540, 4007, 4533, 4597, 4610, 4643, 4676, 5621, 5825, 5831, 5982, 7330, 7350, 7492, 7580, 8170, 8420, 8499, and 8540. Positive picture set 2: 1441, 1610, 1620, 1811, 2035, 2050, 2070, 2091, 2160, 2209, 2224, 2274, 2332, 2345, 2395, 2550, 2650, 4090, 4535, 4599, 4614, 4650, 4668, 5480, 5594, 5700, 5829, 5833, 7200, 7400, 7502, 8185, 8470, 8501, and 8510. Positive picture set 3: 1460, 1630, 1710, 1920, 2040, 2057, 2071, 2150, 2165, 2216, 2300, 2311, 2347, 2388, 2530, 2660, 4255, 4572, 4603, 4622, 4645, 4658, 5260, 5600, 5780, 5830, 5910, 7270, 7470, 8030, 8080, 8210, 8370, 8496, and 8502.
